# Electroacupuncture at the ST36 acupoint increases interleukin-4 responsiveness in macrophages, generation of alternatively activated macrophages and susceptibility to *Leishmania major* infection

**DOI:** 10.1186/1749-8546-7-17

**Published:** 2012-07-28

**Authors:** Danillo N Aguiar, Mayara M Silva, Walki V Parreira, Fernanda D Tome, Lucas F Batista, Clayson M Gomes, Milton AP Oliveira

**Affiliations:** 1 Department of Microbiology, Immunology, Parasitology and Pathology, Tropical Pathology and Public Health Institute, Federal University of Goiás, Goiânia, Goiás, Brazil

## Abstract

**Background:**

Electroacupuncture (EA) has been used to treat inflammatory diseases. Alternatively activated macrophages (AAMo) stimulated by cytokines such as interleukin (IL)-4, IL-10 and IL-13 are anti-inflammatory and mildly microbicidal. This study aimed to evaluate whether EA at the *Zusanli* acupoint (ST36) would change the profile of healthy murine macrophages, particularly the generation of AAMo and susceptibility to *Leishmania major* infection.

**Methods:**

BALB/c mice were treated with EA (15/30 Hz) at the ST36 acupoint for 20 min/d for 5 d. After the final EA session, the mice were euthanized and their peritoneal cells were harvested and counted for determination of arginase activity, nitric oxide (NO) production and microbicidal activity after culture in the presence or absence of IL-4, interferon-γ (IFNγ) or lipopolysaccharide (LPS) or both IFNγ and LPS. Twelve mice were infected with *L. major* promastigotes into the footpads after the final EA session and the infection course was monitored.

**Results:**

Peritoneal cells freshly obtained from EA-treated mice had similar arginase and microbicidal activities to cells from sham-treated mice. After culture with IL-4, cells from EA-treated mice exhibited significant increases in the arginase activity (sham: 58 ± 11.3 *vs.* EA: 80.7 ± 4.6%, *P* = 0.025) and number of parasites/infected cell (sham: 2.5 ± 0.4 *vs.* EA: 4.3 ± 0.8 cells, *P* = 0.007). The NO production was lower in cells from EA-treated mice cultured in the presence of a combination of IFNγ and LPS (sham: 31.6 ± 6.5 *vs.* EA: 22.3 ± 2.1 μM, *P* = 0.025). The lesion size in mice infected with *L. major* promastigotes was larger in EA-treated mice (sham: 3.26 ± 0.29 *vs.* EA: 2.23 ± 0.4 mm, *P* = 0.039).

**Conclusion:**

EA at the ST36 acupoint increases IL-4 responsiveness in macrophages, Generation of AAMo and susceptibility to *L. major* infection

## Background

A number of observations on the anti-inflammatory actions of acupuncture have been published for various acupoints, acupuncture frequencies and additional application of electrostimulation [1]. The insertion of a needle into an acupoint induces the release of pro-inflammatory mediators such as substance P, calcitonin gene-related peptide, histamine, bradykinin, serotonin, proteases, pro-inflammatory cytokines and others, thereby causing vasodilatation and producing danger signals that are transmitted *via* the afferent vagus nerve [1-3]. In response to these stimuli, the hypothalamus secretes corticotropin-releasing hormone (CRH), which leads to a decrease in pro-inflammatory cytokines and an increase in anti-inflammatory cytokines such as interleukin (IL)-10 [3]. Leukocytes also respond to CRH and secrete anti-inflammatory cytokines [4].

It has been shown that electrical stimulation of the ST36 acupoint significantly reduces both the serum and tissue levels of the pro-inflammatory cytokines such as tumor necrosis factor (TNF) in rats with ulcerative colitis [5], chronic inflammation induced by Freund's complete adjuvant [6], experimental arthritis [7,8], inflammation induced by carrageenan injection [9] and other conditions. Furthermore, alternatively activated macrophages (AAMo) are associated with the improvement of several inflammatory diseases, such as experimental arthritis [10] and colitis [11].

Corticoids and IL-10 act on macrophages and increase the generation of AAMo [12,13]. AAMo are mainly induced after stimulation with IL-4 and IL-13, and produces cytokines and enzymes for inflammation modulation and initiation of wound healing [12,13]. The properties of AAMo depend on their arginase activity [14], which increases ornithine and urea production. Ornithine can be metabolized to collagen or purine, which are both fundamental for wound healing as described before [12-14]. In addition, AAMo are more susceptible to intracellular pathogens such as *Leishmania major*[15].

Classically activated macrophages (cMO) are induced by interferon-γ (IFNγ) and produce nitric oxide (NO) through induced NO synthase (iNOS) to enhance the resistance to intracellular pathogens such as *L. major*[13,16]. Therefore, cMO are able to control the growth of intracellular pathogens, while AAMo are susceptible to infection with such pathogens, both *in vitro* and *in vivo*[15-17].

This study aims to evaluate whether electroacupuncture (EA) at the *Zusanli* acupoint (ST36) would change the profile of healthy murine macrophages, particularly the generation of AAMo and susceptibility to *L. major* infection.

## Methods

### Animal***s***

Female BALB/c mice (weighing 18–22 g) were supplied by the Animal Care Facility of the Institute of Tropical Pathology of the Federal University of Goiás, and divided into two groups of three animals each. The experiments were triplicated. The mice were housed under controlled lighting conditions (12-h/12-h light/dark cycle with lights on at 7:00 am) at room temperature with free access to water and food. The Human and Animal Research Committee of the Clinical Hospital of Federal University of Goiás approved all procedures on these animals (Approval Protocol 023/2009).

### EA treatment

Before manipulation with EA, the mice were acclimated to the restraint for one week. The ST36 acupoint is located at the anterior tibial muscle, 5 mm lateral to and below the anterior tubercle of the tibia [18,19]. The mice were treated daily with EA for 5 or 10 d. For the EA treatment, the mice were immobilized in a plastic cylinder that allowed access to the ST36 acupoint. Two stainless-steel needles (0.18 × 8 mm) were inserted (3 mm deep) into the ST36 acupoint. The anode and cathode leads from an electrical stimulator (WQ IOD1; Donghua, China**)** were connected to the two acupuncture needles. The current, a faradic, bipolar and asymmetrical wave with an intensity range of 4–6 mA, was applied for 20 min with increases in a stepwise manner. The stimulation frequency was alternated between 15 and 30 Hz (15/30 Hz). The intensity of the stimulation was determined by the minimum voltage required to cause moderate muscle contraction. The sham group of mice received only needle insertion into a non-acupoint area in the gluteal muscle without electrical stimulation. The mice were euthanized by decapitation immediately after the final EA treatment.

### Peritoneal cells

Resident macrophages were obtained by peritoneal cavity washing with 5 mL of sterile phosphate-buffered saline (PBS). Following aspiration of the fluid, a suspension of the cells was washed twice with PBS and centrifuged at 300 × *g* for 10 min. The suspension was examined in a Neubauer chamber, counted and after adjustment of the concentration to 1 × 10^6^ peritoneal cells for arginase activity analyses and 2 × 10^5^ peritoneal cells for NO and microbicidal assays. The cells were cultured in 24-well plates (TPP, Switzerland) in 0.5 mL of RPMI 1640 medium (Sigma Chemical Co., USA) supplemented with 10% fetal calf serum (FCS; Cripion, Brazil), 2 mM L-glutamine (Sigma Chemical Co. USA), 10 mM Hepes (Sigma Chemical Co. USA), 100 U/mL penicillin (Sigma Chemical Co. USA) and 100 μg/mL streptomycin (Sigma Chemical Co. USA). The cells in the supplemented RPMI medium were incubated at 36°C under 5% CO_2_ in the presence or absence of 1 μg/mL lipopolysaccharide (LPS; *Escherichia coli* O111:B4; Sigma Chemical Co. USA), 5 or 25 ng/mL IL-4 (R&D Systems, USA) or 5 ng/mL IFNγ (R&D Systems, USA). The combination of 1 μg/mL LPS and 2 ng/mL IFNγ was used to evaluate NO production. After 48 h of culture, the supernatant was harvested for the NO analysis, and the cells were subjected to the arginase assay.

### Arginase assay

Peritoneal cells obtained before or after culture were washed twice with PBS and then centrifuged at 300 × *g* for 10 min. The cell pellets (1 × 10^6^ cells) were resuspended in 50 μL of lysis buffer comprising 50 mM Tris–HCl (pH 7.5), 0.1 mM EDTA, 0.1% (v/v) Triton X-100 and 0.5 % (v/v) protease inhibitor cocktail (Sigma Chemical Co., USA). The arginase activity was determined by urea production using a previously described method [20] with some modifications. Briefly, the cell lysate (50 μl) was added to 50 μL of 50 mM Tris–HCl buffer (pH 7.5) containing 10 mM MnCl_2_ (Vetec, Brazil). Macrophage arginase was activated by heating the mixture to 60°C for 10 min. The hydrolysis reaction of L-arginine by arginase was carried out by incubating the lysate with 50 μL of 0.5 M L-arginine (pH 9.7; Sigma Chemical Co., USA) at 37°C for 1 h. The amount of urea production was determined with an enzymatic UREA500® assay kit (Doles, Brazil) according to the manufacturer’s instructions. The results were expressed as mg/dL urea based on a standard curve established with known concentrations of urea.

### Determination of NO production

NO production was estimated by determining the concentration of nitrite in the supernatant of peritoneal cell cultures with or without LPS stimulation using the Griess method [21]. The culture supernatant (100 μL) was incubated with an equal volume of Griess reagent (1.0% sulfanilamide, 0.1% naphazoline hydrochloride, 2.5% orthophosphoric acid; Vetec, Brazil) for 10 min at room temperature. The absorbance at 540 nm was determined using a microplate reader (Multiskan; Thermo Labsystems, Finland). The results were expressed as μM nitrite based on a standard curve established with known concentrations of sodium nitrite (Vetec, Brazil) dissolved in culture medium.

### Mouse infection and assessment of leishmanicidal activity

Promastigotes of *L. major* (clone, MHOM/IL/80/Friedlin; kindly provided by Dr Leda Vieira, Brazil) at 1 × 10^5^ promastigotes/mL were cultured in 24-well plates and passed every 2 d. The culture was performed in Grace’s Insect Medium (Sigma Chemical Co., USA) supplemented with inactivated 20% FCS (Cripion, Brazil), 2 mM L-glutamine (Sigma Chemical Co., USA), 100 U/mL penicillin (Sigma Chemical Co., USA) and 100 μg/mL streptomycin (Sigma Chemical Co., USA). Parasites in the stationary growth phase were harvested at 5 d after the beginning of culture and washed three times in PBS before use. For assessment of leishmanicidal activity, 1 × 10^7^ parasites were injected into the peritoneal cavity of sham- or EA-treated mice. After 3 h, the mice were euthanized by decapitation and peritoneal cells were collected by peritoneal washing with PBS. For removal of polymorphonuclear cells and non-phagocytosed parasites, the cell suspension was centrifuged at 700 × *g* for 15 min at 20°C using Lymphoprep® (Sigma Chemical Co., USA) and washed twice with PBS before culture. Slides of the cells were prepared before and after 48 h of culture in the presence or absence of IL-4 or IFNγ. The slides were stained with an Instant Prov Kit (Newprov, Brazil) for subsequent quantification of the infected cells under a light microscope. In monitoring of the infection course, 1 × 10^6^ parasites were injected into the left footpad of sham- or EA-treated mice. Footpad swelling was measured weekly using a dial thickness gauge (Mitutoyo, Japan), and the thickness increase was calculated as the difference between the left and right footpad measurements.

### Expression of IL-4 receptor in macrophages

Peritoneal cells were washed twice with PBS and resuspended at 2 × 10^5^ cells/50 μL of PBS containing 3% normal goat serum (NGS). A purified anti-IL-4 receptor antibody (1 μL/mL; Genzyme, USA) was added to the cells and incubated for 30 min at 25°C. After two washes with PBS, the cells were resuspended in 50 μL of PBS containing 3% NGS. Next, 5 μL of FITC-conjugated goat anti-rat antibody (Serotec Ltd., UK) was added at 1:10 dilution and the cells were incubated for 20 min at 25°C. The cells were washed twice with PBS and resuspended in 50 μL of PBS containing 3% NGS. After addition of 10 μL of an anti-CD11b APC antibody (0.01 mg/mL; BD Biosciences, USA), the cells were incubated for 20 min at 25°C. Cells were acquired using a C6 Accuri Flow Cytometer (Accuri Cytometers, USA) and analyzed with FCS Express 4 Plus Research Edition software (Denovo Software, USA). The surface expression of IL-4 receptor was quantified as a measure of the mean fluorescence intensity in CD11b^+^ cells.

### Statistical analysis

All experiments were triplicated except the infection course experiments, which were duplicated. Unless specified otherwise, the data were expressed as the mean ± standard deviation (SD) of all replicated experiments. The data for NO production were expressed as the mean ± SD of a representative experiment of the three experiments, owing to high variability in the basal levels. The statistical significance of differences between groups was determined by the Student’s *t*-test (two groups), one-way ANOVA followed by a Tukey test (multiple groups) and two-way ANOVA followed by a Tukey test (infection course curve) using Graph Pad 4.0 software (GraphPad Inc., USA). The results were considered statistically significant at *P* < 0.05.

## Results

### IL-4 responsiveness of peritoneal macrophages

**Figure 1:**
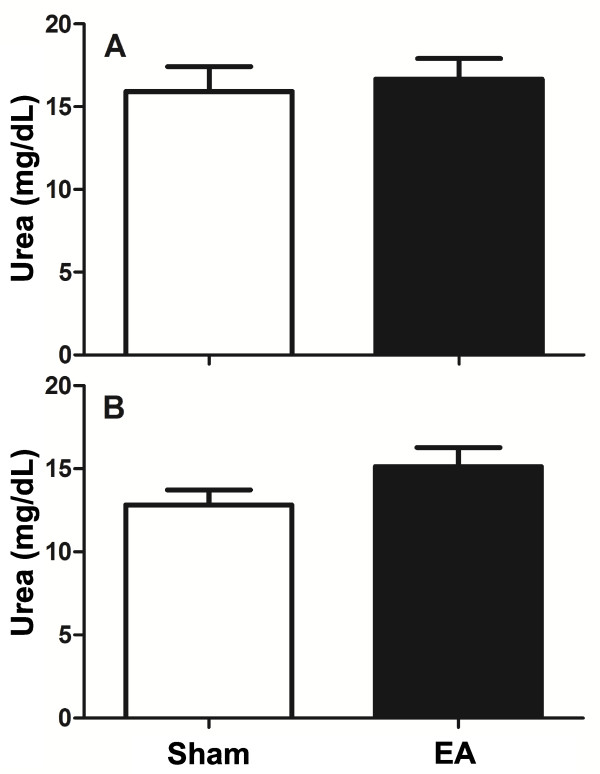
**Arginase activity in cells harvested from the peritoneal cavity of EA-treated mice.** Mice were treated with EA (15/30 Hz) at the Zusanli acupoint (ST36) for 20 min/d for 5 d **(A)** or 10 d **(B)**. After the final EA session, the mice were euthanized and their peritoneal cells were harvested for evaluation by the arginase assay. The data are represented as mean ± SD of urea production per lysate of 1 × 10^6^ cells. The sham-treated mice received only needle insertion into a non-acupoint (gluteal muscle) without electrical stimulation. There were nine mice in each group.

Correct EA treatment and insertion of needles in the ST36 acupoint were recognized by the characteristic rhythmic dorsiflexion of the stimulated hind limb, which was observed in all EA-treated mice. At the end of the final EA treatment, the mice were immediately euthanized and their peritoneal cells were harvested for analysis. The numbers of cells recovered from the peritoneal cavity were similar between the two groups (sham: 2.72 ± 0.5 × 10^6^*vs.* EA: 3.01 ± 0.6 × 10^6^ cells/mouse, *P* = 0.08). The arginase enzyme activities were also similar between the two groups (*P* = 0.34), with urea production ranging from 10.6 to 21.3 mg/dL per 1 × 10^6^ peritoneal cells in each group (Figure 1A). The arginase activities of peritoneal cells were similar for EA- and sham-treated mice for both 5 d (P = 0.34) and 10 d (P = 0.09) (Figure 1A and 1B).

**Figure 2:**
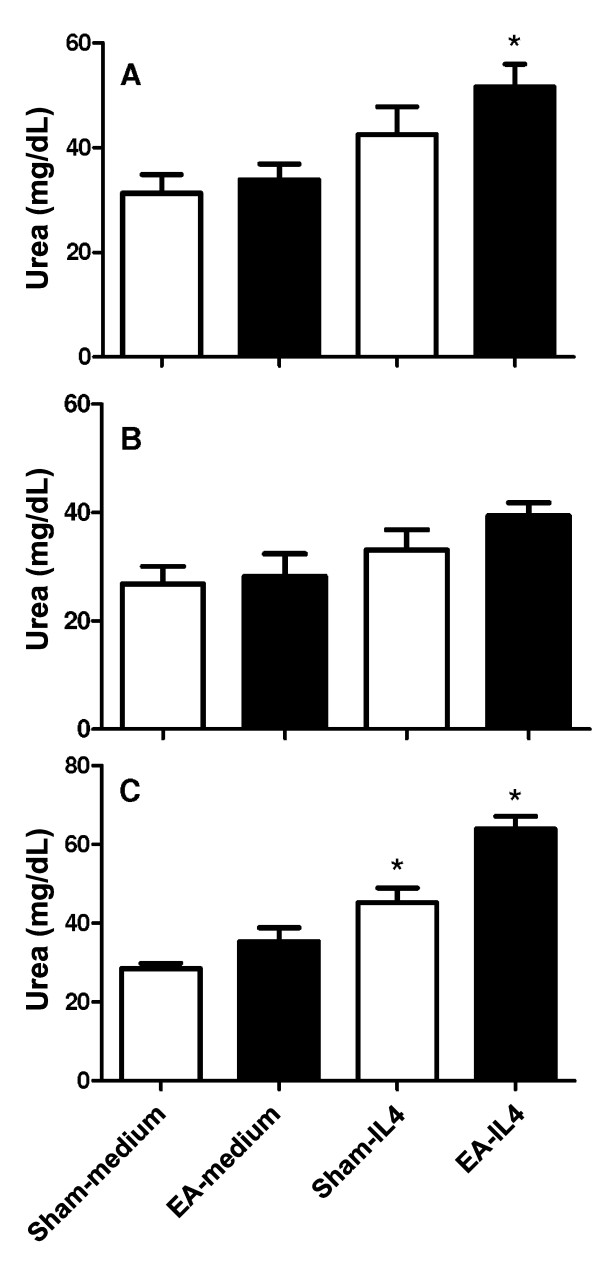
**Arginase activity in cells harvested from the peritoneal cavity of EA-treated mice after culture in the presence of IL-4.** Mice were treated with EA (15/30 Hz) at the Zusanli acupoint (ST36) for 20 min/d for 5 d (A and C) or 10 d **(B)**. After the final EA session, the mice were euthanized and their peritoneal cells were harvested and cultured in the absence (medium) or presence of IL-4 at 5 ng/mL (A and B) or 25 ng/mL **(C)**. After 48 h, the culture medium was discarded, and the cells were lysed for evaluation by the arginase assay. The data are represented as mean ± SD of urea production per lysate of 1 × 10^6^ cells. The sham-treated mice received only needle insertion into a non-acupoint (gluteal muscle) without electrical stimulation. There were nine mice in each group. *P < 0.05, significant difference between sham- and EA-treated mice under the same culture conditions by Student’s *t*-test.

When the peritoneal cells were cultured for 48 h, urea production was increased (reaching 31.3 ± 7.1 mg/dL), indicating an increase in arginase activity. However, the activities in cells from both sham- and EA-treated mice were maintained at similar levels (P = 0.30 and P = 0.41, Figure 2A and B, respectively). Since IL-4 has the ability to promote the generation of AAMo by increase arginase activity [12], we examined whether IL-4 had a different effect on the arginase activity in cells from EA-treated mice. Urea production by peritoneal cells increased from 31.3 ± 7.1 to 42.5 ± 10.7 mg/dL (increase of 35.6%) in cells from sham-treated mice in the presence of IL-4 compared with cells cultured without IL-4 (P = 0.058; Figure 2A). Urea production increased from 33.8 ± 5.9 to 51.6 ± 8.6 mg/dL (increase of 52.2%) in EA-treated mice after IL-4 treatment (P = 0.011; Figure 2A), suggesting an increase of the number of AAMo. Although the increase in IL-4 responsiveness was not significant after 10 d of treatment (P = 0.052), the profile of higher IL-4 responsiveness was maintained in the EA-treated mice (Figure 2B). In another experiment performed with a higher dose of IL-4, urea production was increased by 58% (P = 0.037) in peritoneal cells from sham-treated mice and by 80.7% (P = 0.025) in peritoneal cells from EA-treated mice (Figure 2C). The latter data show that the higher responsiveness to IL-4 was not related to the IL-4 dose.

### NO production

**Figure 3:**
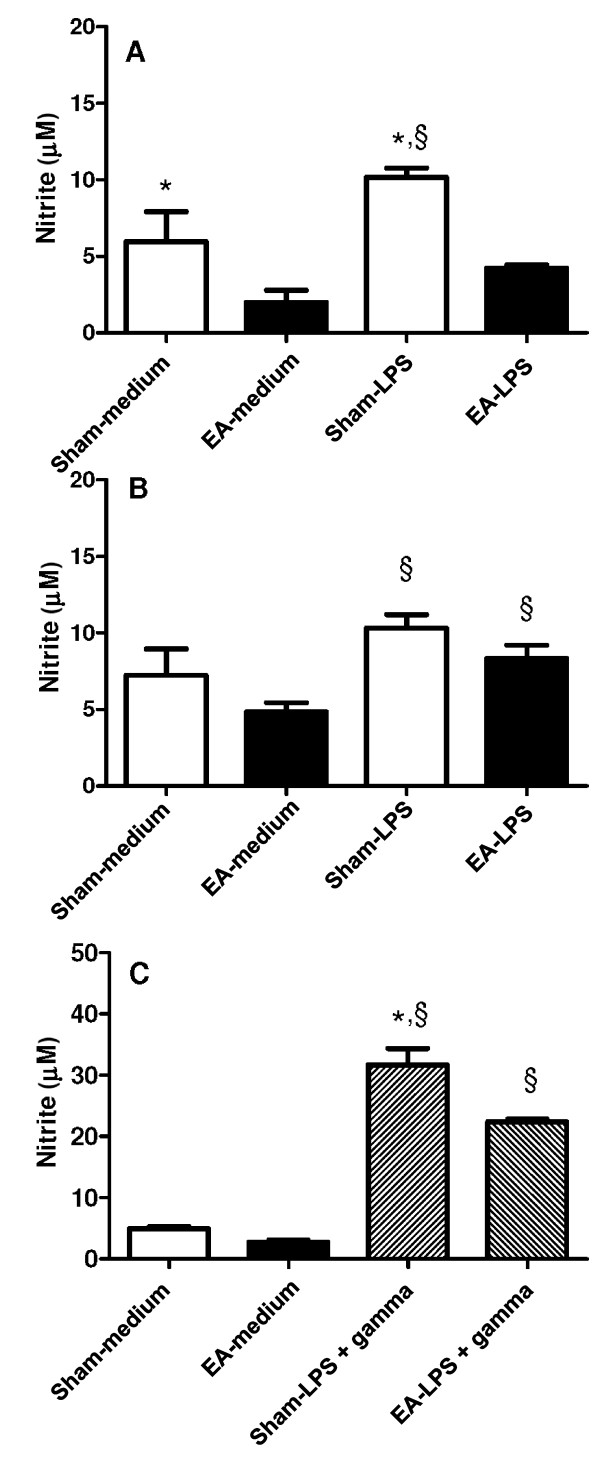
**NO production by cells harvested from the peritoneal cavity of EA-treated mice.** BALB/c mice were treated with EA (15/30 Hz) at the Zusanli acupoint (ST36) for 20 min/d for 5 d (A and C) or 10 d **(B)**. After the final EA session, the mice were euthanized and their peritoneal cells were harvested and cultured in the absence or presence of 1 μg/mL LPS (A and B) or 1 μg/mL LPS and 2 ng/mL IFNγ **(C)**. After 48 h, the culture medium was harvested for nitrite measurement by the Griess assay. The data are represented as mean ± SD of the increase in nitrite production after LPS stimulation. The sham-treated mice received only needle insertion into a non-acupoint (gluteal muscle) without electrical stimulation. The data presented are representative of one of three independent experiments containing three mice in each group. *P < 0.05, significant difference between sham- and EA-treated mice under the same culture conditions by Student’s *t*-test; §P < 0.05, significant difference between cells from the same mouse group under different culture conditions by Student’s *t*-test.

Arginase converts L-arginine to urea, which is the substrate used by iNOS to produce NO in cMO. For evaluation of the NO production by macrophages obtained from EA-treated mice, peritoneal cells were cultured in the presence of LPS or LPS and IFNγ. The NO production by cells from sham-treated mice was significantly higher than the NO production by cells from EA-treated mice (P = 0.046) (Figure 3A). This difference was not significant in the mice treated for 10 d (P = 0.143) (Figure 3B). Although LPS stimulation significantly increased the NO production (sham *vs.* sham-LPS: P = 0.048; EA *vs.* EA-LPS: P = 0.006) (Figure 3B), the classical activation of macrophages depends on IFNγ stimulus [13]. As shown in Figure 3C, IFNγ augmented the NO production stimulated by LPS. The NO production in cells from EA-treated mice was significantly lower than that in cells from sham-treated mice (P = 0.025).

### L. major survival in IL-4-activated peritoneal macrophages

**Figure 4:**
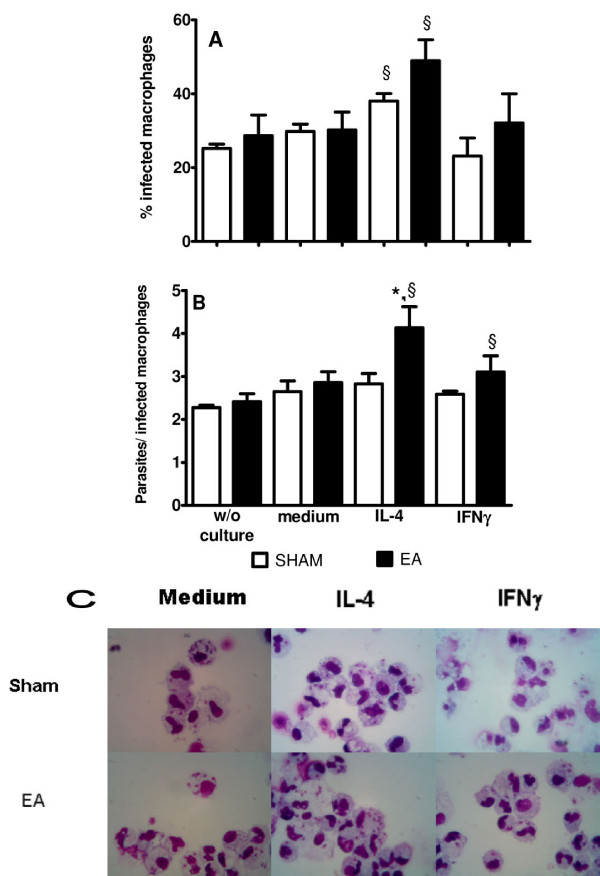
**Leishmanicidal activity of cells harvested from the peritoneal cavity of EA-treated mice.** Mice were treated with EA (15/30 Hz) at the Zusanli acupoint (ST36) for 20 min/d for 5 d. After the final EA session, the mice were inoculated intraperitoneally with 1 × 10^7^ L. major promastigotes. At 3 h after infection, the mice were euthanized and their peritoneal cells were harvested. Their mononuclear cells were then purified with Lymphoprep® solution. Slides of cells were prepared before culture (w/o culture) and after 48 h of culture in the absence (medium) or presence of 5 ng/mL IL-4 or 5 ng/mL IFNγ. The slides were stained with the Instant Prov Kit and analyzed under a light microscope. The data are represented as mean ± SD of the percentages of infected cells (A) or numbers of parasites/infected cell **(B)**. Panel **C** shows cells after 48 h of culture under different conditions. The sham-treated mice received only needle insertion into a non-acupoint (gluteal muscle) without electrical stimulation. There were nine mice in each group. *P < 0.05, significant difference between sham- and EA-treated mice in the same culture conditions by one-way ANOVA followed by the Tukey test; §P < 0.05, significant difference between cells from the same mouse group under different culture conditions by one-way ANOVA followed by the Tukey test.

Activation of iNOS or arginase in macrophages is essential for controlling the growth of the intracellular parasite L. major. Since our data suggested that EA favors the activation of arginase over iNOS, we investigated whether macrophages obtained from EA-treated mice had a decreased ability to kill L. major. Mice were injected intraperitoneally with live L. major promastigotes, and euthanized after 3 h to obtain peritoneal cells. The phagocytosis of parasites by macrophages obtained at 3 h after L. major injection was similar between sham- and EA-treated mice (P = 0.271; Figure 4A, w/o culture). Culture of the infected cells for 48 h without IL-4 or IFNγ led to similar percentages of infected cells and amounts of parasites inside the macrophages between the macrophages from sham- and EA-treated mice (P = 0.84 and P = 0.601, respectively). However, there was an increase in the percentage of infected macrophages in the presence of IL-4 (sham vs. sham-IL-4: P = 0.013; EA vs. EA-IL-4: P = 0.029; Figure 4A). Macrophages from EA-treated mice stimulated with IL-4 showed an increase in the number of parasites per infected cell (sham: 2.5 ± 0.4 vs. EA: 4.3 ± 0.8; P = 0.007; Figure 4B), suggesting that these macrophages were more susceptible to L. major infection. The presence of parasites inside the macrophages is depicted in Figure 4C.

### Expression of IL-4 receptor in macrophages

**Figure 5:**
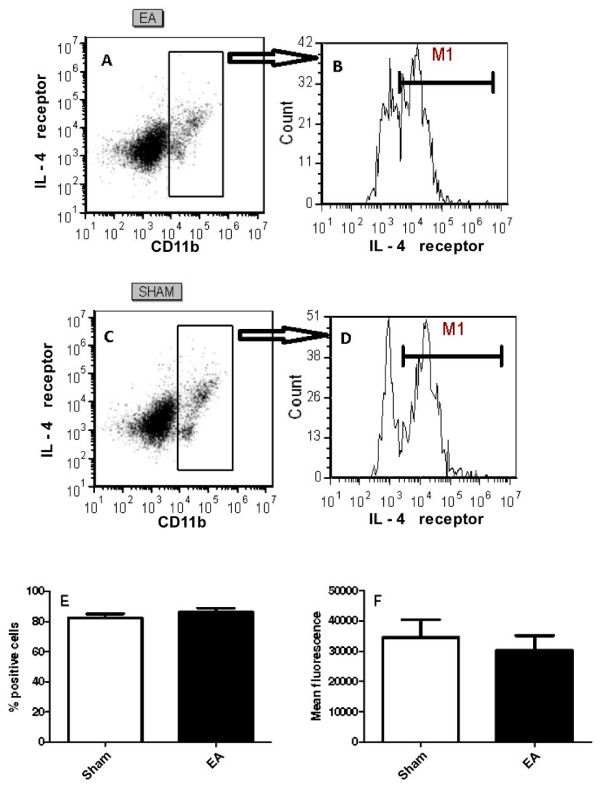
**IL-4 receptor expression in peritoneal macrophages after EA treatment.** Mice were treated with EA (15/30 Hz) at the Zusanli acupoint (ST36) for 20 min/d for 5 d. After the final EA session, the mice were euthanized and their peritoneal cells were harvested and stained for CD11b and IL-4 receptor. **(A and C)** Dot blot analyses of total peritoneal cells. **(B and D)** IL-4 receptor expression in CD11b^+^ cells. The percentages of IL-4 receptor ^+^ cells in CD11b^+^ cells **(E)** and mean fluorescence intensity of IL-4 receptor expression in CD11b^+^ cells **(F)** are also shown for three independent experiments containing three mice in each group. The sham-treated mice received only needle insertion into a non-acupoint (gluteal muscle) without electrical stimulation.

Since the IL-4 responsiveness was increased in macrophages after EA, we investigated IL-4 receptor expression in the peritoneal cells from sham- or EA-treated mice. IL-4 receptor expression was detected in 80% of macrophages (CD11b^+^) from both groups (Figure 5B, D and E), with no significant difference in the percentages of expression between the two groups (P = 0.273). The mean fluorescence intensities of the IL-4 receptor expression in CD11b^+^ cells were similar in the sham- and EA-treated mice (P = 0.515).

**Figure 6:**
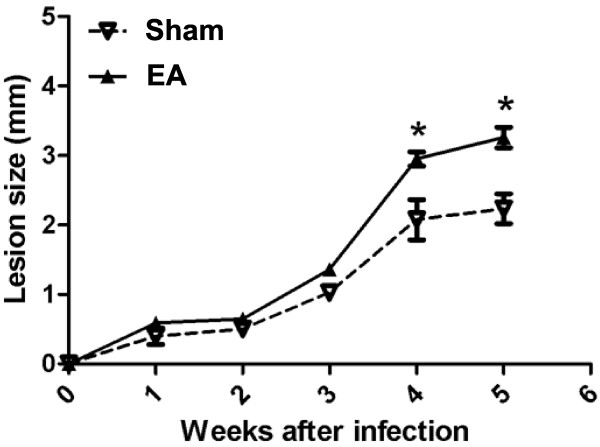
**Lesions developed by sham- or EA-treated mice after L. major infection.** BALB/c mice were treated with EA (15/30 Hz) at the Zusanli acupoint (ST36) for 20 min/d for 5 d. After the final EA session, the mice were infected with 2 × 10^6^ stationary-phase promastigotes of L. major into one of the hind footpads. The lesion size was calculated as the difference between the left and right footpad measurements. The sham-treated mice received only needle insertion into a non-acupoint (gluteal muscle) without electrical stimulation. The data are represented as mean ± SD of two independent experiments containing three mice in each group. *P < 0.05, significant difference between sham- and EA-treated mice by two-way ANOVA followed by a Tukey test.

### EA increases L. major survival in vivo

To verify whether the increased survival of parasites also occurred in vivo after EA treatment, we treated the mice with EA for 5 d and then immediately injected with promastigote parasites into the footpad at the end of the final EA treatment. The lesion size was significantly higher in EA-treated mice than in sham-treated mice (P = 0.039).

## Discussion

Macrophages have well-established roles in the primary responses to pathogens, and can be activated in different ways, giving rise to cMO or AAMo [12,14]. Arginase is the prototypic marker for AAMo [12,14]. This enzyme increases in murine macrophages involved in helminthic infection [14], tumors [22] and tissue repair [23], converting L-arginine to ornithine and urea, while iNOS present in cMO converts L-arginine to citrulline and NO [12]. Arginase and iNOS share the same substrate, leading to an inverse correlation between these two enzymes in helminthic infection, tumors and tissue-repair environments [14,22,23]. In our experiments, EA suppressed the NO production induced by LPS or LPS and IFNγ in peritoneal macrophages. Although the arginase activity in cells after EA treatment or cultured in the absence of IL-4 did not change, the activity was increased in macrophages from EA-treated mice in the presence of IL-4. It is not clear how EA increases the IL-4 responsiveness of macrophages, although EA and IL-4 could synergistically potentiate the responsiveness or interfere with some signal transduction from the IL-4 receptor. However, the mechanism is unlikely due to an increase in IL-4 receptor expression, because the IL-4 receptor expression was similar between macrophages from sham- and EA-treated mice.

The generation of AAMo with high arginase activity is associated with a decreased ability to control intracellular parasites such as L. major, while NO production by cMO is associated with better control of these parasites [15]. Experiments have been performed using IFNγ-stimulated macrophages to observe the killing of L. major in vitro. In addition, IL-4 can prevent NO production and decrease the killing of the parasites [15,24]. These findings are in agreement with our results, showing an increase in the percentage of cells infected by L. major after IL-4 treatment. Macrophages from EA-treated mice were more responsive to IL-4 and enhanced the growth of the parasite inside the cells. An ability of IFNγ-stimulated macrophages to kill parasites was not observed in our experiments, possibly because the combination of LPS and IFNγ is required to produce the optimal stimulation for NO production to kill parasites [24], and only IFNγ was used in some of our experiments.

Infection of BALB/c mice with L. major induces early production of IL-4 by CD4^+^ T cells carrying the Vβ4α8 T cell receptor at 16 h after infection [25]. This early production of IL-4 and the presence of AAMo are associated with the susceptibility of this mouse strain to L. major infection [15,25]. Since macrophages from EA-treated mice were more responsive to IL-4, the outcome of infecting mice with L. major was examined. The lesion size in EA-treated mice was more pronounced than that in sham-treated mice, suggesting that EA increases IL-4 responsiveness in vivo and interferes with the outcome of infection by intracellular pathogens such as L. major.

Electrical stimulation was not used in the sham-treated mice in our experiments. The experiments could rule out the effects of electrical current because of the small size of the mice. Electrical stimulation outside of an acupoint could travel to a nearby acupoint to produce effects with different intensities. The properties of acupoints include increased conductance and reduced impedance and resistance [26], and minimal electrical stimulation of an acupoint could provide significant effects in sham-treated mice [27].

Significant effects of EA were only observed when the mice were treated for 5 d. The decreased effects of EA in the mice treated for 10 d could be caused by tolerance. EA tolerance was observed in rats treated with a polarized current for 30 min/d for 6 d at the frequency of 100 Hz [28,29].

The anti-inflammatory properties of the ST36 acupoint have been examined in previous studies [5-9]. Our findings demonstrated that EA increased the IL-4 responsiveness of macrophages. In addition, our experiments showed a decrease in the microbicidal activity associated with the generation of AAMo.

## Conclusion

EA at the ST36 acupoint in mice increases the IL-4 responsiveness of macrophages, generation of AAMo and susceptibility to L. major infection.

## Abbreviations

EA, Electroacupuncture; AAMo, Alternatively activated macrophages; ST36, Zusanli acupoint; NO, Nitric oxide; IL, Interleukin; IFNγ, Interferon-γ; LPS, Lipopolysaccharide; CRH, Corticotropin-releasing hormone; TNF, Tumor necrosis factor; PBS, Phosphate-buffered saline; cMO, Classically activated macrophages; iNOS, Induced nitric oxide synthase; NGS, Normal goat serum; SD, Standard deviation.

## Competing interests

The authors declare that they have no competing interests.

## Authors’ contributions

DNA and MAPO conceived and designed the study. DNA performed the acupuncture. MMS, LFB and FDT performed the evaluations of arginase activity and nitric oxide production. WVP and CMG performed the evaluation of leishmanicidal activity in vitro and in vivo. All authors read and approved the final manuscript.

## References

[B1] Zijlstra FJ van den Berg-de Lange I Huygen FJ Klein J Anti-inflammatory actions of acupuncture Mediators Inflamm 2003 12 59 69 10.1080/0962935031000114943 12775355 PMC1781596

[B2] Kavoussi B Ross BE The neuroimmune basis of anti-inflammatory acupuncture Integr Cancer Ther 2007 6 251 257 10.1177/1534735407305892 17761638

[B3] Cabioglu MT Cetin BE Acupuncture and immunomodulation Am J Chin Med 2008 36 25 36 10.1142/S0192415X08005552 18306447

[B4] Cho ZH Hwang SC Wong EK Son YD Kang CK Park TS Bai SJ Kim YB Lee YB Sung KK Neural substrates, experimental evidences and functional hypothesis of acupuncture mechanisms Acta Neurol Scand 2006 113 370 377 10.1111/j.1600-0404.2006.00600.x 16674603

[B5] Tian L Huang YX Tian M Gao W Chang Q Downregulation of electroacupuncture at ST36 on TNF-alpha in rats with ulcerative colitis World J Gastroenterol 2003 9 1028 1033 12717850 10.3748/wjg.v9.i5.1028 PMC4611366

[B6] Wang WJ Lu J Niu CS Huang YR Ma Q YG A Hao HW Li LM Tu Y [Effects of electroacupuncture of unilateral and bilateral "zusanli" (ST 36) on serum TNF-alpha, IL-1 and IL-4 levels in rats with chronic inflammatory pain] Zhen Ci Yan Jiu 2008 35 429 432 21375016

[B7] He TF Yang WJ Zhang SH Zhang CY Li LB Chen YF Electroacupuncture inhibits inflammation reaction by upregulating vasoactive intestinal Peptide in rats with adjuvant-induced arthritis Evid Based Complement Alternat Med 2011 290489 10.1155/2011/290489 PMC2952296 20953422

[B8] Fang JQ Shao XM Ma GZ Effect of electroacupuncture at "Zusanli" (ST 36) and "Sanyinjiao" (SP 6) on collagen-induced arthritis and secretory function of knee-joint synoviocytes in rats Zhen Ci Yan Jiu 2009 34 93 96 19685721

[B9] Chae Y Hong MS Kim GH Hahm DH Park HJ Ha E Kim MJ Yang J Lee H Protein array analysis of cytokine levels on the action of acupuncture in carrageenan-induced inflammation Neurol Res 2007 29 Suppl 1 S55 S58 17359642 10.1179/016164107X172365

[B10] Takahashi N de Jager VC Gluck A Letzkus M Hartmann N Staedtler F Ribeiro-Dias F Heuvelmans-Jacobs M van den Berg WB Joosten LA The molecular signature of oxidative metabolism and the mode of macrophage activation determine the shift from acute to chronic disease in experimental arthritis: critical role of interleukin-12p40 Arthritis Rheum 2008 58 3471 3484 10.1002/art.23956 18975327

[B11] Hunter MM Wang A Parhar KS Johnston MJ Van Rooijen N Beck PL McKay DM In vitro-derived alternatively activated macrophages reduce colonic inflammation in mice Gastroenterology 2010 138 1395 1405 10.1053/j.gastro.2009.12.041 20044996

[B12] Martinez FO Helming L Gordon S Alternative activation of macrophages: an immunologic functional perspective Annu Rev Immunol 2009 27 451 483 10.1146/annurev.immunol.021908.132532 19105661

[B13] Zhang X Mosser DM Macrophage activation by endogenous danger signals J Pathol 2008 214 161 178 10.1002/path.2284 18161744 PMC2724989

[B14] Maizels RM Pearce EJ Artis D Yazdanbakhsh M Wynn TA Regulation of pathogenesis and immunity in helminth infections J Exp Med 2009 206 2059 2066 10.1084/jem.20091903 19770272 PMC2757871

[B15] Kropf P Fuentes JM Fahnrich E Arpa L Herath S Weber V Soler G Celada A Modolell M Muller I Arginase and polyamine synthesis are key factors in the regulation of experimental leishmaniasis in vivo FASEB J 2005 19 1000 1002 15811879 10.1096/fj.04-3416fje

[B16] Liew FY O'Donnell CA Immunology of leishmaniasis Adv Parasitol 1993 32 161 259 8237615 10.1016/s0065-308x(08)60208-0

[B17] Biswas A Bhattacharya A Kar S Das PK Expression of IL-10-triggered STAT3-dependent IL-4Ralpha is required for induction of arginase 1 in visceral leishmaniasis Eur J Immunol 2011 41 992 1003 10.1002/eji.201040940 21413004

[B18] Kim SK Lee Y Cho H Koo S Choi SM Shin MK Hong MC Min BI Bae H A Parametric Study on the Immunomodulatory Effects of Electroacupuncture in DNP-KLH Immunized Mice Evid Based Complement Alternat Med 2011 389063 10.1093/ecam/nep166 PMC3135419 19900958

[B19] Yim YK Lee H Hong KE Kim YI Lee BR Son CG Kim JE Electro-acupuncture at acupoint ST36 reduces inflammation and regulates immune activity in Collagen-Induced Arthritic Mice Evid Based Complement Alternat Med 2007 4 51 57 10.1093/ecam/nel054 17342241 PMC1810363

[B20] Corraliza IM Campo ML Soler G Modolell M Determination of arginase activity in macrophages: a micromethod J Immunol Methods 1994 174 231 235 10.1016/0022-1759(94)90027-2 8083527

[B21] Green SJ Crawford RM Hockmeyer JT Meltzer MS Nacy CA Leishmania major amastigotes initiate the L-arginine-dependent killing mechanism in IFN-gamma-stimulated macrophages by induction of tumor necrosis factor-alpha J Immunol 1990 145 4290 4297 2124240

[B22] Munder M Arginase: an emerging key player in the mammalian immune system Br J Pharmacol 2009 158 638 651 10.1111/j.1476-5381.2009.00291.x 19764983 PMC2765586

[B23] Daley JM Brancato SK Thomay AA Reichner JS Albina JE The phenotype of murine wound macrophages J Leukoc Biol 2010 87 59 67 10.1189/jlb.0409236 20052800 PMC2801619

[B24] Holscher C Arendse B Schwegmann A Myburgh E Brombacher F Impairment of alternative macrophage activation delays cutaneous leishmaniasis in nonhealing BALB/c mice J Immunol 2006 176 1115 1121 16394000 10.4049/jimmunol.176.2.1115

[B25] Launois P Maillard I Pingel S Swihart KG Xenarios I Acha-Orbea H Diggelmann H Locksley RM MacDonald HR Louis JA IL-4 rapidly produced by V beta 4 V alpha 8 CD4+ T cells instructs Th2 development and susceptibility to Leishmania major in BALB/c mice Immunity 1997 6 541 549 10.1016/S1074-7613(00)80342-8 9175832

[B26] Ahn AC Martinsen OG Electrical characterization of acupuncture points: technical issues and challenges J Altern Complement Med 2007 13 817 824 10.1089/acm.2007.7193 17983337 PMC2386953

[B27] Lundeberg T Lund I Sing A Naslund J Is Placebo Acupuncture What It is Intended to Be? Evid Based Complement Alternat Med 2011 932407 10.1093/ecam/nep049 PMC3139519 19525330

[B28] Tian JH Zhang W Fang Y Xu W Grandy DK Han JS Endogenous orphanin FQ: evidence for a role in the modulation of electroacupuncture analgesia and the development of tolerance to analgesia produced by morphine and electroacupuncture Br J Pharmacol 1998 124 21 26 10.1038/sj.bjp.0701788 9630338 PMC1565350

[B29] Ulett GA Han S Han JS Electroacupuncture: mechanisms and clinical application Biol Psychiatry 1998 44 129 138 10.1016/S0006-3223(97)00394-6 9646895

